# Genetic risk factors for restenosis after percutaneous coronary intervention in Kazakh population

**DOI:** 10.1186/s40246-016-0077-z

**Published:** 2016-06-08

**Authors:** Elena V. Zholdybayeva, Yerkebulan A. Talzhanov, Akbota M. Aitkulova, Pavel V. Tarlykov, Gulmira N. Kulmambetova, Aisha N. Iskakova, Aliya U. Dzholdasbekova, Olga A. Visternichan, Dana Zh. Taizhanova, Yerlan M. Ramanculov

**Affiliations:** National Center for Biotechnology, 13/5, KorgalzhinskoeHighway, Astana, Kazakhstan; National Scientific Medical Research Center, 42 Abylai Khan Avenue, Astana, Kazakhstan; Karaganda State Medical University, 40, Gogol Street, Karaganda, Kazakhstan; School of Science and Technology, Nazarbayev University, 53 Kabanbay Batyr Ave, Astana, Kazakhstan; Al-Farabi Kazakh National University, Almaty, Kazakhstan

**Keywords:** Coronary heart disease, Restenosis, SNP, Genotyping

## Abstract

**Background:**

After coronary stenting, the risk of developing restenosis is from 20 to 35 %. The aim of the present study is to investigate the association of genetic variation in candidate genes in patients diagnosed with restenosis in the Kazakh population.

**Methods:**

Four hundred fifty-nine patients were recruited to the study; 91 patients were also diagnosed with diabetes and were excluded from the sampling. DNA was extracted with the salting-out method. The patients were genotyped for 53 single-nucleotide polymorphisms. Genotyping was performed on the QuantStudio 12K Flex (Life Technologies). Differences in distribution of BMI score among different genotype groups were compared by analysis of variance (ANOVA). Also, statistical analysis was performed using R and PLINK v.1.07. Haplotype frequencies and LD measures were estimated by using the software Haploview 4.2.

**Results:**

A logistic regression analysis found a significant difference in restenosis rates for different genotypes. *FGB* (rs1800790) is significantly associated with restenosis after stenting (OR = 2.924, *P* = 2.3E−06, additive model) in the Kazakh population. *CD14* (rs2569190) showed a significant association in the additive (OR = 0.08033, *P* = 2.11E−09) and dominant models (OR = 0.05359, *P* = 4.15E−11). *NOS3* (rs1799983) was also highly associated with development of restenosis after stenting in additive (OR = 20.05, *P* = 2.74 E−12) and recessive models (OR = 22.24, *P* = 6.811E−10).

**Conclusions:**

Our results indicate that *FGB* (rs1800790), *CD14* (rs2569190), and *NOS3* (rs1799983) SNPs could be genetic markers for development of restenosis in Kazakh population. Adjustment for potential confounder factor BMI gave almost the same results.

## Background

Coronary heart disease (CHD) is a disease characterized by reduced blood supply to the heart muscle. Narrowing of the coronary arterial lumen due to atherosclerosis is the primary cause in 97–98 % of CHD cases. Coronary heart disease has the highest rate of death and serious complications among all forms of cardiovascular disease. An estimated 17.5 million people died from cardiovascular diseases in 2012, representing 31 % of all global deaths. Of these deaths, an estimated 7.4 million were due to CHD and 6.7 million were due to stroke [1]. It should be noted that CHD usually affects the population aged between 35 and 65 years. In addition, CHD represents the most important cause of sudden cardiac deaths. Together with cerebrovascular diseases, CHD accounts for 64 % of all cardiovascular deaths [2].

According to the Ministry of Healthcare and Social Development of the Republic of Kazakhstan prior to year 2012, CHD morbidity has been constantly increasing in 2009–2012 with exception of 2013 when a minor decrease in the morbidity was observed [3]. In 2013, there were 59,799 new cases of CHD registered in Kazakhstan and the morbidity rate reached 500.6 cases per 100,000 population, compared to 445.6 cases in 2011 and 507.4 cases in 2012 [3].

Advances in medicine have led to the emergence of novel methods of CHD treatment, such as angioplasty or coronary stenting. The first use of coronary stenting in clinical practice was in 1986 [4]. Primary percutaneous coronary intervention (PCI) has become a well-established strategy for patients with coronary heart disease [5]. Nowadays, endovascular methods for the reestablishment of coronary blood flow preserve the lives and health of hundreds of thousands of people around the world. Nevertheless, there is a possibility that during the first 6 months to 1 year after successful coronary stenting, a symptomatic relapse of angina may occur due to the development of restenosis. Reoccurrence of stenosis is a major limitation to the effectiveness of the stenting, and even the use of drug-eluting stents does not solve the problem completely [6]. After coronary stenting, the restenosis rate is 20–30 %. The use of second generation drug-eluting stents has reduced this rate, but the development of restenosis after implantation remains a serious clinical problem [7].

Restenosis can occur for many different reasons. The pathophysiological mechanisms of restenosis have not yet been fully explained, but it is believed that those mechanisms include inflammation, proliferation, and matrix remodeling. Over the years, many predictive clinical, biological, genetic, epigenetic, lesion-related, and procedural risk factors for restenosis have been identified. Those factors are useful in the risk stratification of patients and also contribute to our understanding of this condition [8]. In this sense, the search for new predictor factors in the development of restenosis is topical.

Currently, the genetic factors of restenosis have been studied mostly in European populations. The ethnical variability of genetic markers is well known, as shown in the results of the GENetic DEterminants of Restenosis (GENDER) study discussed in the article by Verschuren et al. The GENDER databank contains the genotypic data of 2,571,586 single-nucleotide polymorphism (SNPs) from 295 cases with restenosis and 571 matched controls. The set that included all 36 reported genes in the literature was indeed significantly associated with restenosis in the GENDER study (*P* = 0.024). Subsequent analyses of the individual genes demonstrated that this association was determined by 6 of the 36 genes [9]. As a result of the GENDER study, the selected SNPs have been associated with the risk of developing restenosis in European populations.

Based on literature review, candidate genes for restenosis were not studied or validated in Kazakh population. That is why the purpose of the current study is to look for associations of genetic variation in candidate genes in patients diagnosed with restenosis after percutaneous coronary intervention in the Kazakh population.

## Methods

### Study population

There were initially 459 patients with diagnosed CHD recruited to the study. Of these patients, 91 were also diagnosed with diabetes and were excluded from the sampling, since several studies have shown that diabetes is an independent risk factor for restenosis and may introduce bias to the interpretation of results [10]. Anthropometrical and biochemical characteristics were gathered for the population sample comprising 368 patients (299 males and 69 females). There were 99 case subjects, those who manifested in-stent restenosis within 6 months after stenting and 269 control subjects and those who did not developed restenosis after stenting. The study protocol was approved by the Ethics Committee of the National Center for Biotechnology. All subjects were ethnic Kazakhs.

### Genotyping

Whole blood samples of 368 patients (9 ml) were collected into tubes containing 50 mmol/l ethylenediaminetetraacetic acid (disodium salt). DNA was extracted with the salting-out method. [11]. Genotyping of the extended panel of polymorphisms of candidate genes was performed on the QuantStudio 12K Flex (Life Technologies). The total reaction volume was 5 μl, containing 2.5 μl of 2× OppenArray Real-time master mix, and 2.5 μl of DNA concentration of 50 ng/μl. Temperature conditions were 10 min at 93 °C; cycling for 45 s at 93 °C, 13 s at 94 °C, and 2.14 min at 53.5 °C for 50 cycles, followed by incubation at 25 °C for 2 min. Data analysis was performed using the software package TaqMan Genotyper Software v.1.3.

Table 1 shows a panel of 53 SNPs used in the current study. All SNPs were selected based on the results of the GENDER study.

**Table 1:** Description of SNPs included in the study

Gene	Polymorphism	Locus
Adrenergic beta-2-receptor (ADRB2)	rs1042713	5q31–q32
Advanced glycosylation end product-specific receptor (AGER)	rs2070600	6p21.3
Advanced glycosylation end product-specific receptor (AGER)	rs1800624	6p21.3
Angiotensin II receptor, type 1 (AGTR1)	rs5186	3q24
Angiotensin II receptor, type 1 (AGTR1)	rs5182	3q24
Butyrylcholinesterase (BCHE)	rs1803274	3q26.1–q26.2
Chemokine (C–C motif) ligand 11 (CCL11)	rs4795895	17q21.1–q21.2
Cluster of differentiation 14 (CD14)	rs2569190	5q31.1
Cyclin-dependent kinase inhibitor 1B (p27, Kip1, CDKN1B)	rs34330	12p13.1–p12
Collagen, type III, alpha 1 (Col3A1)	rs1800255	2q31
Colony stimulating factor 2 (CSF2)	rs25882	5q31.1
Chemokine (C-X3-C motif) receptor 1 (CX3CR1)	rs3732379	3p21.3
Cytochrome b-245, alpha polypeptide (CYBA)	rs4673	16q24
Cytochrome P450, family2, subfamily C, polypeptide 19 (CYP2C19)	rs12248560	10q24
Fibrinogen beta chain (FGB)	rs1800790	4q28
Fibrinogen beta chain (FGB)	rs1044291	4q28
Coagulation factor V (F5)	rs6025	1q23
Glutathione peroxidase 1 (GPX1)	rs8179164	3p21.3
Integrin, beta 2 (ITGB2)	rs235326	21q22.3
Lipoprotein lipase (LPL)	rs328	8p22
Matrix metallopeptidase 12 (MMP12)	rs12808148	11q22.3
Matrix metallopeptidase 12 (MMP12)	rs17099726	20q11.2–q13.1
Matrix metallopeptidase 12 (MMP12)	rs2276109	20q11.2–q13.1
Methylenetetrahydrofolate reductase (NAD(P)H) MTHFR)	rs1801133	1p36.3
Nitric oxide synthase 3 (NOS3)	rs2070744	7q36
Nitric oxide synthase 3 (NOS3)	rs1799983	7q36
K(lysine) acetyltransferase2B (KAT2B, PCAF)	rs2948080	3p24
K(lysine) acetyltransferase2B (KAT2B, PCAF)	rs6776870	3p24
K(lysine) acetyltransferase2B (KAT2B, PCAF)	rs2929404	3p24
K(lysine) acetyltransferase2B (KAT2B, PCAF)	rs17796904	3p24
Peroxisome proliferator-activated receptor gamma (PPARG)	rs3856806	3p25
C-ros oncogene1, receptor tyrosine kinase (ROS1)	rs529038	6q22
Thrombomodulin (THBD)	rs1042579	20p11.2
Thrombospondin 4 (THBS4)	rs1866389	5q13
Thrombopoietin (THPO)	rs6141	3q27
Tumor protein p53 (TP53)	rs1042522	17p13.1
Transferrin (TF)	rs1799899	3p
Uncoupling protein 3 (UCP3)	rs1800849	11q13.4
Vitamin D receptor (VDR)	rs11568820	12q13.11
Vitamin D receptor (VDR)	rs11574027	12q13.11
Vitamin D receptor (VDR)	rs11574077	12q13.11
Tumor necrosis factor α (TNFα)	rs1800629	6p21.3
Tumor necrosis factor α (TNFα)	rs361525	6p21.3
Interleukin 1 receptor antagonist (IL1RN)	rs419598	2q14.2
Interleukin 1α (IL1A)	rs1800587	2q12–q21
Interleukin 1 β (IL1B)	rs1143627	2q13–q21
Interleukin 4 (IL4)	rs2243250	5q23–31
Interleukin 6 (IL6)	rs1800796	7p21
Interleukin 8 (IL8)	rs 4073	4q12–q13
Interleukin 10 (IL10)	rs1800871	1q31–q32
Interleukin 10 (IL10)	rs1800872	1q31–q32
Interleukin 10 (IL10)	rs1800896	1q31–q32
Interleukin 10 (IL10)	rs3024498	1q31–q32

### Statistical analysis

A pairwise correlation matrix was made to check for multicollinearity between measured variables. A stepwise regression was performed to evaluate significance of potential confounders. A logistic regression analysis with adjustment for potential confounder was used to test for differences between statuses of restenosis according to genotyping. Associations between each SNP and development of the restenosis were tested according to three genetic models: additive (cumulative effect of each additional variant allele), dominant (homozygous wild-type vs. variant allele-carrying genotype) and recessive (wild-type allele carrying genotype vs. homozygous variant genotype). Every SNP that reached statistically significant level of *P* < 0.0001 were reported. Significant SNPs were further annotated using RegulomeDB that harbors information for known and predicted regulatory elements [12]. Annotation of functional variation in personal genomes using RegulomeDB. Additionally, a differences in distribution of BMI score among different genotype groups were compared by one-way analysis of variance (one-way ANOVA). For ANOVA test a default threshold of 0.05 was used to report significance. Statistical analysis was performed using R and PLINK [13, 14].

LD statistical analysis was performed using Haploview 4.2. For block generations, Hardy-Weinberg *P* value cutoff 0.001 was used [15]. We ignored SNPs that minor allele frequencies (MAF) of less than 0.001. For block generations, the confidence intervals default algorithm was used.

## Results

A total of 368 patients participated in the study. Body mass index (BMI), the blood levels of cholesterol, low-density lipoproteins (LDL), and high-density lipoproteins (HDL) were recorded for every patient at the time of participation. Table 2 shows summary statistics of the measured variables.

**Table 2:** Anthropometrical and biochemical characteristics of the population sample (*n* = 368)

Restenosis (case/control)	99/269
Gender (male/female)	299/69
Age (years)	58.61 ± 11.67
BMI (kg/m^2^)	28.35 ± 4.53
Cholesterol (mmol/l)	4.93 ± 1.24
LDL (mmol/l)	3.05 ± 1.18
HDL (mmol/l)	1.24 ± 0.63

*BMI* body mass index, *LDL* low-density lipoproteins, *HDL* high-density lipoproteins

Anthropometrical and measured biochemical characteristics for potential confounders were evaluated before performing a test for association between genotype distribution and restenosis status. The distributions of the measured traits fitted the normality assumption and were included in the analysis as is without any transformation. Construction of the correlation matrix revealed that in our dataset, cholesterol and LDL are highly correlated with each other (Table 3). In order to avoid multicollinearity, cholesterol was excluded from further analysis.

**Table 3:** Pairwise correlation matrix of measured variables

	BMI	HDL	LDL	Cho	Age
Age	−0.072	−0.067	−0.061	−0.081	1
Cho	0.402	0.364	0.739	1	
LDL	0.322	0.492	1		
HDL	0.163	1			
BMI	1				

The remaining traits were used to build a statistical model that predicts the development of restenosis as a main outcome. Age, LDL, and HDL contribute insignificantly to restenosis variability in our dataset based on the results from stepwise regression analysis. The final statistical model included BMI as a single confounding factor that may affect the association between genotype and restenosis status.

There were 368 patients available to collect whole blood for further genetic analysis. After applying quality control filters, the final dataset contained 268 patients with genotype information for 48 SNPs. Four SNPs were excluded based on the Hardy Weinberg equilibrium test (*P* ≤ 0.001). One SNP was excluded because of its low genotyping rate.

SNPs that reached a significant level *P* < 0.0001 were reported. The results from logistic regression revealed that *FGB* (rs1800790) SNP was associated with restenosis after stenting. Notably that homozygous A allele carriers of the rs1800790 SNP are 24 times less likely to develop restenosis after stenting compared to other allele carries. *CD14* (rs2569190) SNP showed highly significant correlation development of restenosis. Based on results, carrying homozygous G allele is a risk factor for development of restenosis. Finally, a missense mutation rs1799983 SNP mapped to *NOS3* was also highly associated with the development of restenosis after stenting. Both additive and recessive models showed similar results, suggesting that carrying additional G allele for rs1799983 SNP is a protective factor against developing restenosis. Summary for the results of logistic regression analysis is shown in Table 4.

**Table 4:**
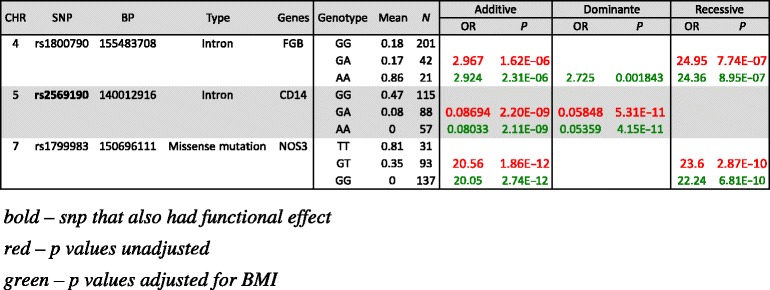
Results from logistic regression analysis

*Bold*snp that also had functional effect, *red P* values unadjusted, *green P* values adjusted for BMI

Association of genotype of the candidate genes 53 SNP with the restenosis was examined with one-way ANOVA by comparing the mean scores of BMI to the genotype. According to ANOVA, BMI distribution had significant differences with genotypes of rs6025 SNP in coagulation factor V also known as *F5* (*P* = 0.00643) in control group. rs419598 SNP mapped to *IL1RN* was positively associated with BMI in the control group (*P* = 0.0299). Finally, rs4795895 SNPs mapped to *CCL11* gene showed significant differences with BMI in group of patients (*P =* 0.012).Genotypes of other SNPs were not significantly different.

**Fig. 1:**
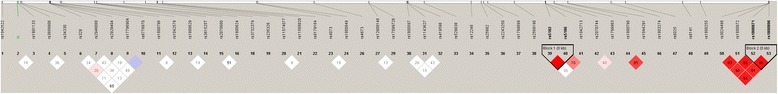
LD SNP plot. The LD is displayed according to standard color schemes, with *bright red* for very strong LD (LOD > 2, D’ = 1), *light red* (LOD > 2, D’ < 1) and *blue* (LOD < 2, D’ = 1) for intermediate LD, and *white* (LOD < 2, D’ < 1) for no LD

Using Haploview 4.2 software, LD statistics results for the Kazakh population were obtained (Fig. 1) (HW *P* cutoff, 0.001; MAF, 0.001). As a result, two haplotype blocks were defined: one block consisting of two SNPs, i.e., rs5182 and rs5186 (block 1, chromosome 3); one block consisting of two SNPs, i.e., rs1800871 and rs1800896 (block 2, chromosome 1). The haplotype frequencies in the studied population are presented in Table 5. Haplotype CC (rs5182-rs5186, block 1) was associated with the risk of developing restenosis (OR 2.17; 95 % CI; 1.33–3.53, *P* = 0.002) and the haplotype GC (rs1800871-rs1800896, block 2) was associated with restenosis (OR 1.51; CI, 1.01–2.26, *P* = 0.04).

**Table 5:** Frequencies (%) of *AGTR* haplotypes [rs5182(573C>T), rs5186 (1166A>C)] and *IL10* haplotypes [rs1800871(−819 C>T), and rs1800896 (−1082 A>G)] in patient with and without restenosis

		With restenosis (*n* = 99)	Without restenosis (*n* = 269)	OR	95 % CI	*P* value
Locus	Haplotype	Hf	Hf			
Block1	TA	0.634	0.669	–	–	NS
rs5182|rs5186	CA	0.162	0.221	–	–	NS
573C>T|1166A>C	CC	0.205	0.110	2.17	1.33–3.53	0.002
Block 2	AT	0.455	0.493	–	–	NS
rs1800871|rs1800896	GC	0.340	0.238	1.51	1.01–2.26	0.04
819 C>T|−1082 A>G	GT	0.198	0.266	–	–	NS

## Discussion

The present study included Kazakhs that are Turkic people of the northern parts of Central Asia (largely Kazakhstan). From the historic point of view and because of scarce genetic data, it was concluded that Kazakh population was formed as a result of admixture of the European and Asian populations [16]. Case and controls were diagnosed with CHD. BMI was increased in both groups. Our results showed that BMI is a single confounding factor that may affect the association between genotype and restenosis status. In turn, genotype polymorphisms of *F5*, *IL1RN*, *CCL11* genes significantly influence on increased body mass index.

The present study focuses on 48 SNPs in 36 candidate genes that were previously reported as genetic risk factors for restenosis. Association of hemostatic gene polymorphisms with restenosis after coronary stent placement was the first genetic risk to be described [17, 18]. The association of selected SNPs in inflammation-related genes with restenosis is also well documented [19–21]. In addition, a number of candidate genes in the renin-angiotensin hormone system and the endothelial nitric oxide synthase (*eNOS*, Glu298Aps and −786T\C) are also involved in this process [22–24]. New molecular markers of restenosis are constantly emerging. For example, in a recent study, SNPs in *VDR* (vitamin D-dependent receptor) gene were considered as risk markers of restenosis [25]. Fragoso et al. reported that transforming growth factor-1β (rs1800469) was associated with the risk of developing restenosis after coronary stenting in Mexican patients [26]. This SNP was not investigated in our study.

Our study has revealed that blood coagulation fibrinogen factor I (*FGB*), monocyte differentiation antigen CD14 (*CD14*), and nitric oxide synthase 3 (*NOS3*) genes are among the factors associated with the risk of restenosis in the studied population.

Monocytes play a central role in restenosis after balloon angioplasty and stent implantation. Monocytes migrate into the damaged area either as a direct response or through the release of platelet-derived factors. Activated monocytes release large amounts of proinflammatory cytokines, which cause vasoconstriction and non-specific recruitment, proliferation, and activation of other cells including vascular smooth muscle cells in the vascular wall. The activation of monocytes/macrophages, endothelial cells, and smooth muscle cells mediated by CD14 and/or CD14 may play an important role in the restenosis processes [27].

Functional C(-260)→T polymorphism in the promoter of the *CD14* gene has been reported to be associated with CHD but data have yielded conflicting results. In the meta-analysis of Zhang et al., TT genotype is associated with ischemic heart disease in the East Asian population but not in the European or Indian populations [28]. Two previous studies have investigated the role of CD14 in the development of restenosis, one being a prospective study by Zee et al. [29] in 779 patients and the other being a prospective study by Shimada et al. [27] in 129 Japanese patients. They found the −260T/T genotype to be a risk factor for restenosis. But the GENDER study found that T allele was not associated with restenosis in the European population [9]. Also, it was showed that the CD14+CD16+CX3CR1+monocytes might have a role in-stent restenosis following coronary implantation of bare-metal stents in patients with acute myocardial infarction [30].

The results of our study have revealed that *CD14* promoter polymorphism remained statistically significant in the additive (OR = 0.08033, *P* = 2.11E−09) and dominant models (OR = 0.05359, *P* = 4.15E−11). Therefore, the T allele at position −260 of *CD14* gene is a risk allele for restenosis in Kazakh population. Population stratification based on ethnicities may lead to inconsistency, especially when both allele frequencies and incidence rates of the diseases vary across ethnic groups.

Fibrinogen (factor I) is a glycoprotein synthesized by the liver. It consists of three polypeptides Aα, Bβ, and γ coded by the alpha (*FGA*), beta (*FGB*), and gamma (*FGG*) genes, respectively. Fibrinogen is an important component of the coagulation cascade and a major determinant of blood viscosity and platelet aggregation [31]. Polymorphisms of *FGB* was shown to be associated with coronary heart disease [32, 33]. In a study by Völzke in 2004, there was no association between the β-fibrinogen −455G/A and the risk of restenosis after PTCA or recurrent restenosis after re-PTCA [34]. In the GENDER study, a multicenter prospective study, the association of gene polymorphism *FGB* −455G>A(rs1800790) with the risk of restenosis was not found in the European population [9]. In the study by Oguri in 2007, the association of *FGB* −455G>A (rs1800790) with restenosis was shown in the Japanese population [35]. Our results suggest that genotype *FGB* −455G>A (rs1800790) is significantly associated with restenosis after stenting (OR = 2.924, *P* = 2.3E−06, additive model) in the Kazakh population. Interestingly that the same SNP in dominant model gave close *P* value, but much higher OR = 24.36, suggesting that allele A is a recessive protective factor against development of restenosis. Thus, it can be concluded that the relationship between genetic polymorphisms of *FGB −455G>A* (rs1800790) and the development of restenosis is not universal between different ethnic groups. It is also interesting to observe that Japanese and Kazakhs are alike in turns of effect from polymorphisms of *FGB −455G>A* (rs1800790).

Nitric oxide synthase 3 (NOS3) locates at chromosome 7q36, and it encodes endothelial nitric oxide synthase (eNOS), which can generate nitric oxide (NO) in endothelial cells. Endothelial NO is a key determinant of vascular homeostasis, and it can also participate in vascular repair. Dysfunction of any of these processes may result in atherosclerotic and thrombotic diseases [36–38]. The association between several polymorphisms of the *NOS3* gene and CAD and restenosis risks has been previously studied [39–41]. In the meta-analysis by Zhang et al., 18 case-control studies with 2994 cases and 3130 controls, including 13 studies of East-Asia descendents, 5 studies of non-East-Asian descendents indicated that *eNOS* 894G/T polymorphism may play an important role in CHD development among Asian population [42]. In 2010, Li et al. performed a meta-analysis involving 20 studies relating non-Asian population and 3 studies relating Asian population and found significant association of T allele in *eNOS* 894G/T with CHD in non-Asian population [43]. Other studies showed that homozygosity of Glu298Asp and 786T/C polymorphisms of the *NOS3* gene represented an independent risk factor for in-stent restenosis, and the 894G/T polymorphism of *NOS3* gene was associated with an increased risk of death and/or myocardial infarction within 1 year after stent placement [24, 44, 45]. Shuvalova et al. showed that minor allele of polymorphism 298G/T of the *eNOS* gene (rs1799983) is associated with an increased risk of in-stent restenosis [46].

A number of polymorphisms have been identified in the *NOS3* gene among which two polymorphisms in the promoter region (−786C/T), and one in the exon (894G/T or Glu298Asp) was studied. Only one of these polymorphisms *NOS3* (rs1799983) has been found to be significantly associated with restenosis in our study OR = 20.05, *P* = 2.74E−12 in additive and OR = 22.24, *P* = 6.81E−10 in recessive models. Thus, the present results demonstrated that there might be a significant association between the *NOS3* polymorphism (rs1799983) and restenosis after PCI in the Kazakh population.

In the linkage disequilibrium analysis, *AGTR1* risk (CC) haplotype and *IL10* (GC) for developing restenosis were detected in our study. Su et al. studied 16 *AGTR1* polymorphisms. Based on the linkage disequilibrium pattern among these SNPs, six polymorphisms were selected as haplotype tagging SNPs and further were genotyped. SNP analyses indicated that GTC haplotype (rs275650, rs2276736, rs5182) associated with the risk of developing myocardial infarction [47]. Koch et al. investigated the possibility that single-nucleotide polymorphisms of the genes encoding *TNF* (_863C/A, _308G/A), *LT-a* (252G/A), and *IL10* (_1082G/A, _819C/T, and _592C/A) are associated with the incidence of restenosis, death, or myocardial infarction (MI) after coronary stenting [48]. With regard to the *IL10* polymorphisms, they observed three different haplotypes, _1082G/_819C/_592C (GCC), ACC, and ATA, with relative frequencies of 0.45, 0.29, and 0.26, respectively. Koch et al. have not detected a significant correlation between restenosis and the frequency of the haplotypes [48]. But in our study, it was identified that GC haplotype of *IL1*0 (819 C>T, −1082 A>G) was associated with restenosis. Thus, individuals with the risk haplotype have compromised function of inflammatory reactions.

## Conclusion

In conclusion, the present study examined the association between 48 SNPs and restenosis in the Kazakh population. Our results indicate that BMI is a single confounding factor and *FGB* (rs1800790), *CD14* (rs2569190), and *NOS3* (rs1799983). SNPs could be genetic markers for the development of restenosis in the Kazakh population. Genotyping of these polymorphisms may be used in predicting the risk of restenosis in the Kazakh population.

## Abbreviations

ANOVA, analysis of variance; BMI, body mass index; CHD, coronary heart disease; GENDER, GENetic DEterminants of Restenosis; HDL, high-density lipoproteins; LDL, low-density lipoproteins, PCI, percutaneous coronary intervention; SNP, single-nucleotide polymorphism

## References

[1] World Health Organization. Cardiovascular diseases. http://www.who.int/mediacentre/factsheets/fs317/en/. Accessed 9 February 2016.

[2] Tardif J-C (2010). Coronary artery disease in 2010. Eur Heart J.

[3] Medical statistics [In Russian]. http://www.medinfo.kz/medstat.jsp. Accessed 9 February 2016.

[4] Sigwart U, Puel J, Mirkovitch V, Joffre F, Kappenberger L (1987). Intravascular stents to prevent occlusion and restenosis after transluminal angioplasty. N Engl J Med.

[5] Fischman D, Leon M, Baim D, Schatz R, Savage M, Penn I, Detre K, Veltri L, Ricci D, Nobuyoshi M (1994). A randomized comparison of coronary-stent placement and balloon angioplasty in the treatment of coronary artery disease. Stent Restenosis Study Investigators. N Engl J Med.

[6] Spertus J, Nerella R, Kettlekamp R, Marso S, Borkon A, Rumsfeld J, House J (2005). Risk of restenosis and health status outcomes for patients undergoing percutaneous coronary intervention versus coronary artery bypass graft surgery. Circulation.

[7] Otsuki S, Sabate M (2014). Drug-eluting stents and acute myocardial infarction: a lethal combination or friends?. World J Cardiol.

[8] Jukema J, Verschuren J, Ahmed T, Quax P (2011). Restenosis after PCI. Part 1: pathophysiology and risk factors. Nat. Rev. Cardiol..

[9] Verschuren J, Trompet S, Postmus I, Sampietro M, Heijmans B, Houwing-Duistermaat J, Slagboom P, Jukema J (2012). Systematic testing of literature reported genetic variation associated with coronary restenosis: results of the GENDER Study. PLoS One.

[10] Mercado N, Boersma E, Wijns W, Gersh B, Morillo C, de Valk V, van Es G, Grobbee D, Serruys P (2001). Clinical and quantitative coronary angiographic predictors of coronary restenosis: a comparative analysis from the balloon-to-stent era. J Am Coll Cardiol.

[11] Miller S, Dykes D, Polesky H (1988). A simple salting out procedure for extracting DNA from human nucleated cells. Nucleic Acids Res..

[12] Boyle AP, Hong EL, Hariharan M, Cheng Y, Schaub MA, Kasowski M, Karczewski KJ, Park J, Hitz BC, Weng S, Cherry JM, Snyder M (2012). Annotation of functional variation in personal genomes using RegulomeDB. Genome Res.

[13] The R project for statistical computing. https://www.r-project.org/. Accessed 9 February 2016.

[14] Purcell S, Neale B, Todd-Brown K, Thomas L, Ferreira MA, Bender D, Maller J, Sklar P, de Bakker PI, Daly MJ, Sham PC (2007). PLINK: a tool set for whole-genome association and population-based linkage analyses. Am J Hum Genet.

[15] Barrett JC, Fry B, Maller J, Daly MJ (2005). Haploview: analysis and visualization of LD and haplotype maps. Bioinformatics (Oxford, England).

[16] Comas D, Calafell F, Mateu E, Perez-Lezaun A, Bosch E, Martinez-Arias R, Clarimon J, Facchini F, Fiori G, Luiselli D, Pettener D, Bertranpetit J (1998). Trading genes along the silk road: mtDNA sequences and the origin of central Asian populations. Am J Hum Genet.

[17] Kastrati A, Dirschinger J, Schomig A (2000). Genetic risk factors and restenosis after percutaneous coronary interventions. Herz.

[18] Ortlepp J, Hoffmann R, Killian A, Lauscher J, Merkelbach-Brese S, Hanrath P (2001). The 4G/5G promotor polymorphism of the plasminogen activator inhibitor-1 gene and late lumen loss after coronary stent placement in smoking and nonsmoking patients. Clin Cardiol.

[19] Toutouzas K, Colombo A, Stefanadis C (2004). Inflammation and restenosis after percutaneous coronary interventions. Eur Heart J.

[20] Monraats P, Kurreeman F, Pons D, Sewgobind V, de Vries F, Zwinderman A, de Maat M, Doevendans P, de Winter R, Tio R, Waltenberger J, Huizinga T, Eefting D, Quax P, Frants R, van der Laarse A, van der Wall E, Jukema J (2007). Interleukin 10: a new risk marker for the development of restenosis after percutaneous coronary intervention. Genes Immun.

[21] Tashiro H, Shimokawa H, Sadamatsu K, Aoki T, Yamamoto K (2001). Role of cytokines in the pathogenesis of restenosis after percutaneous transluminal coronary angioplasty. Coron Artery Dis.

[22] Ryu S, Cho E, Park H, Im E, Jang Y, Shin G, Shim W, Cho S (2002). Renin-angiotensin-aldosterone system (RAAS) gene polymorphism as a risk factor of coronary in-stent restenosis. Yonsei Med J.

[23] Wijpkema JS, van Haelst PL, Monraats PS, Bruinenberg M, Zwinderman AH, Zijlstra F, van der Steege G, de Winter RJ, Doevendans PA, Waltenberger J, Jukema JW, Tio RA (2006). Restenosis after percutaneous coronary intervention is associated with the angiotensin-II type-1 receptor 1166A/C polymorphism but not with polymorphisms of angiotensin-converting enzyme, angiotensin-II receptor, angiotensinogen or heme oxygenase-1. Pharmacogenet Genomics.

[24] Gomma A, Elrayess M, Knight C, Hawe E, Fox K, Humphries S (2002). The endothelial nitric oxide synthase (Glu298Asp and -786 T > C) gene polymorphisms are associated with coronary in-stent restenosis. Eur Heart J.

[25] Monraats P, Fang Y, Pons D, Pires N, Pols H, Zwinderman A, de Maat M, Doevendans P, DeWinter R, Tio R, Waltenberger J, Frants R, Quax P, van der Laarse A, van der Wall E, Uitterlinden A, Jukema J (2010). Vitamin D receptor: a new risk marker for clinical restenosis after percutaneous coronary intervention. Expert Opin Ther Targets.

[26] Fragoso JM, Zuniga-Ramos J, Arellano-Gonzalez M, Alvarez-Leon E, Villegas-Torres BE, Cruz-Lagunas A, Delgadillo-Rodriguez H, Pena-Duque MA, Martinez-Rios MA, Vargas-Alarcon G (2015). The T29C (rs1800470) polymorphism of the transforming growth factor-beta1 (TGF-beta1) gene is associated with restenosis after coronary stenting in Mexican patients. Exp Mol Pathol.

[27] Shimada K, Miyauchi K, Mokuno H, Watanabe Y, Iwama Y, Shigekiyo M, Matsumoto M, Okazaki S, Tanimoto K, Kurata T, Sato H, Daida H (2004). Promoter polymorphism in the CD14 gene and concentration of soluble CD14 in patients with in-stent restenosis after elective coronary stenting. Int J Cardiol.

[28] Zhang HF, Zhong BL, Zhu WL, Xie SL, Qiu LX, Zhu LG, Wang Y, Lei L (2009). CD14 C-260 T gene polymorphism and ischemic heart disease susceptibility: a HuGE review and meta-analysis. Genet Med.

[29] Zee RY, Hoh J, Cheng S, Reynolds R, Grow MA, Silbergleit A, Walker K, Steiner L, Zangenberg G, Fernandez-Ortiz A, Macaya C, Pintor E, Fernandez-Cruz A, Ott J, Lindpainter K (2002). Multi-locus interactions predict risk for post-PTCA restenosis: an approach to the genetic analysis of common complex disease. Pharmacogenomics J.

[30] Liu Y, Imanishi T, Ikejima H, Tsujioka H, Ozaki Y, Kuroi A, Okochi K, Ishibashi K, Tanimoto T, Ino Y, Kitabata H, Akasaka T (2010). Association between circulating monocyte subsets and in-stent restenosis after coronary stent implantation in patients with ST-elevation myocardial infarction. Circ J.

[31] Scott EM, Ariens RA, Grant PJ (2004). Genetic and environmental determinants of fibrin structure and function: relevance to clinical disease. Arterioscler Thromb Vasc Biol.

[32] Reiner AP, Carty CL, Carlson CS, Wan JY, Rieder MJ, Smith JD, Rice K, Fornage M, Jaquish CE, Williams OD, Tracy RP, Lewis CE, Siscovick DS, Boerwinkle E, Nickerson DA (2006). Association between patterns of nucleotide variation across the three fibrinogen genes and plasma fibrinogen levels: the Coronary Artery Risk Development in Young Adults (CARDIA) study. J Thromb Haemost.

[33] Martiskainen M, Pohjasvaara T, Mikkelsson J, Mantyla R, Kunnas T, Laippala P, Ilveskoski E, Kaste M, Karhunen PJ, Erkinjuntti T (2003). Fibrinogen gene promoter -455 A allele as a risk factor for lacunar stroke. Stroke.

[34] Volzke H, Grimm R, Robinson DM, Wolff B, Schwahn C, Hertwig S, Motz W, Rettig R (2004). Candidate genetic markers and the risk of restenosis after coronary angioplasty. Clin Sci.

[35] Oguri M, Kato K, Hibino T, Yokoi K, Segawa T, Matsuo H, Watanabe S, Nozawa Y, Murohara T, Yamada Y (2007). Identification of a polymorphism of UCP3 associated with recurrent in-stent restenosis of coronary arteries. Int J Mol Med.

[36] Voetsch B, Jin RC, Loscalzo J (2004). Nitric oxide insufficiency and atherothrombosis. Histochem Cell Biol.

[37] Palmer RM, Ashton DS, Moncada S (1988). Vascular endothelial cells synthesize nitric oxide from L-arginine. Nature.

[38] Radomski MW, Palmer RM, Moncada S (1987). The anti-aggregating properties of vascular endothelium: interactions between prostacyclin and nitric oxide. Br J Pharmacol.

[39] Galluccio E, Piatti P, Citterio L, Lucotti PC, Setola E, Cassina L, Oldani M, Zavaroni I, Bosi E, Colombo A, Alfieri O, Casari G, Reaven GM, Monti LD (2008). Hyperinsulinemia and impaired leptin-adiponectin ratio associate with endothelial nitric oxide synthase polymorphisms in subjects with in-stent restenosis. Am J Physiol Endocrinol Metab.

[40] Antoniades C, Tousoulis D, Vasiliadou C, Pitsavos C, Chrysochoou C, Panagiotakos D, Tentolouris C, Marinou K, Koumallos N, Stefanadis C (2005). Genetic polymorphism on endothelial nitric oxide synthase affects endothelial activation and inflammatory response during the acute phase of myocardial infarction. J Am Coll Cardiol.

[41] Cam SF, Sekuri C, Tengiz I, Ercan E, Sagcan A, Akin M, Berdeli A (2005). The G894T polymorphism on endothelial nitric oxide synthase gene is associated with premature coronary artery disease in a Turkish population. Thromb Res.

[42] Zhang K, Bai P, Shi S, Zhou B, Wang Y, Song Y, Rao L, Zhang L (2012). The G894T polymorphism on endothelial nitric oxide synthase gene is associated with increased coronary heart disease among Asia population: evidence from a Meta analysis. Thromb Res.

[43] Li J, Wu X, Li X, Feng G, He L, Shi Y (2010). The endothelial nitric oxide synthase gene is associated with coronary artery disease: a meta-analysis. Cardiology.

[44] Suzuki T, Okumura K, Sone T, Kosokabe T, Tsuboi H, Kondo J, Mukawa H, Kamiya H, Tomida T, Imai H, Matsui H, Hayakawa T (2002). The Glu298Asp polymorphism in endothelial nitric oxide synthase gene is associated with coronary in-stent restenosis. Int J Cardiol.

[45] Gorchakova O, Koch W, von Beckerath N, Mehilli J, Schomig A, Kastrati A (2003). Association of a genetic variant of endothelial nitric oxide synthase with the 1 year clinical outcome after coronary stent placement. Eur Heart J.

[46] Shuvalova YA, Kaminnyi AI, Meshkov AN, Shirokov RO, Samko AN (2012). Association between polymorphisms of eNOS and GPx-1 genes, activity of free-radical processes and in-stent restenosis. Mol Cell Biochem.

[47] Su S, Chen J, Zhao J, Huang J, Wang X, Chen R, Gu D (2004). Angiotensin II type I receptor gene and myocardial infarction: tagging SNPs and haplotype based association study. The Beijing atherosclerosis study. Pharmacogenetics.

[48] Koch W, Tiroch K, von Beckerath N, Schomig A, Kastrati A (2003). Tumor necrosis factor-alpha, lymphotoxin-alpha, and interleukin-10 gene polymorphisms and restenosis after coronary artery stenting. Cytokine.

